# Chromosomal polymorphisms have no negative effect on reproductive outcomes after IVF/ICSI-ET/FET

**DOI:** 10.1038/s41598-022-20132-8

**Published:** 2022-11-09

**Authors:** Jing Zhao, Bixia Huang, Jie Hao, Bin Xu, Yanping Li

**Affiliations:** 1 Reproductive Medicine Center, Xiangya Hospital, Central South University, 87 Xiangya Road, Changsha City, Hunan Province People’s Republic of China; 2 Clinical Research Center for Women’s Reproductive Health in Hunan Province, Changsha, Hunan People’s Republic of China

**Keywords:** Genetics, Reproductive disorders

## Abstract

The present study aimed to explore whether chromosomal polymorphisms (CPs) have negative effects on reproductive outcomes of in vitro fertilization/intracytoplasmic sperm injection-embryo transfer (IVF/ICSI-ET)/frozen-thawing embryo transfer (FET)? We conducted a retrospective study consisting of 21,867 assisted reproductive technology treatment cycles, among which, fresh embryo transfer cycles accounted for 10,400, and the rest were FET cycles. According to karyotype of CPs, the former was grouped as: group 1 (male carrier, n = 425), group 2 (female carrier, n = 262), and group 3 (couple without CPs, n = 9713). Accordingly, FET cycles were divided into 3 groups: group 4 (male carrier, n = 298), group 5 (female carrier, n = 311), and group 6 (couple without CPs, n = 10,858). The embryo implantation rate (IR), clinical pregnancy rate (CPR), live birth rate (LBR), and early miscarriage rate (EMR) were compared among the groups. In fresh embryo transfer cycles after IVF/ICSI, there were no significant differences in the infertility duration, BMI, basal FSH, no. of oocyte, no. of 2PN, endometrial thickness on trigger day, serum E2, P, and LH level on trigger day (*P* > 0.05). The female age, no. of 2PN embryo cleavage, top-quality embryo, and no. of embryo transferred were significantly different among groups (*P* < 0.05). The IR was 38.8%, 36.2%, and 34.0% in groups 1, 2, and 3, respectively. The CPR was 55.1%, 52.3%, and 49.7%, respectively. The LBR was 36.9%, 37.4%, and 36.4%, respectively. The CPR and LBR showed no significant differences among groups. The IR was lower and the EMR was higher in group 3 than those of groups 1 and 2. Binary logistic regression analysis indicated that female age, no. of embryo transferred, EMT, LH, and P on the trigger day were independently factors associated with CPR. Besides, no. of embryo transferred, and EMT on trigger day were associated with LBR, while the CPs was not related with CPR and LBR after IVF/ICSI-ET. In FET cycles, the infertility duration was similar (*P* > 0.05), but the female age, BMI, no. of embryo transferred were significantly different among groups (*P* > 0.05). The IR was 24.3%, 23.6% and 22.3% in group 4, 5, and 6, receptivity. The CPR was 31.8%, 30.9%, and 30.0%, the LBR was 23.8%,26.3%, and 23.8%, while the EMR was 12.6%, 13.1%, 14.4%, respectively. The IR, CPR, EMR, and LBR showed no significant differences among groups (*P* > 0.05). Binary logistic regression analysis indicated that female age, infertility duration, and no. of embryo transferred were independently factors affecting CPR and LBR after FET. The CPs were not associated with CPR and LBR after FET. The results suggested that uniparental carrying of CPs have no effects on the reproductive outcomes after IVF/ICSI-ET/FET. However, it is not clear whether both parents carrying CPs would affect pregnancy outcome.

## Introduction

Chromosomal polymorphisms (CPs) are defined as the heritable variants of segments located in heterochromatic chromosomal regions, including variations in heterochromatic segments, satellites and satellite stalks^1^. On non-acrocentric chromosomes, CPs typically occur in the heterochromatic regions of the long arms of chromosomes 1, 9, and 16 in human, as well as in the distal heterochromatic region of the Y chromosome (qh+)^2^. For acrocentric chromosomes, such as those in D and G groups, variations occur usually on satellites, satellite stalks, or short arms^3^. In addition, pericentric inversions on chromosomes 1, 2, and 9 are also considered as polymorphisms^1,4^.

The incidence of CPs is about 2–5% in the general population^5^. Because heterochromatic regions are rich in highly repetitive satellite DNA sequences, and with no coding proteins, CPs are generally regarded as harmless variants and have no functional or phenotypic effects on the CPs carriers^6^.

However, these “harmless” regions seem to be associated with many reproductive abnormalities. Recently, an increasing number of researches have shown an increased incidence of CPs variations in infertile couples^7^, recurrent pregnancy loss (RPL) or spontaneous miscarriages^8,9^.

Assisted reproductive technology (ART) was an effective treatment for infertility. Some reports suggested there were bad impacts of CPs on ART outcomes, while the others showed that the CPs had no negative effects on the pregnancy outcomes after IVF/ICSI-ET^10,11^. Studies by Li and Xu et al. showed that both male and female carriers have adverse impacts on reproductive outcomes after fresh IVF/ICSI-ET cycles^6,12^. But there were also some studies reporting that only male carriers of CPs had poor pregnancy outcomes after IVF/ICSI-ET treatment^4,6,13–15^. However, aforementioned studies only included fresh IVF/ICSI-ET cycles. Therefore, the impact of CPs on IVF/ICSI/FET outcomes is still controversial, and its impacts on pregnancy outcomes of FET were unclear.

To further explore the associations between CPs and ART endpoints, especially pregnancy outcomes in FET, we conducted this retrospective study. This research might gain our understanding of reproductive outcomes after IVF/ICSI-ET/FET in CPs carriers.

## Materials and methods

### Subjects

The retrospective study was conducted using the data of infertile couples undergoing fresh IVF/ICSI-ET or FET treatments from January 2010 to December 2021 in the Reproductive Medicine Center, Xiangya Hospital, Central South University. The present research was approved by the ethical committee of Xiangya Hospital, Central South University. and was exempted from informed consent requirements owing to its retrospective design.

The inclusion criteria were as follows: age 20–40 years; BMI 18–30 kg/m^2^; 3 U/L < bFSH < 10 U/L; infertile couples ever underwent karyotype test. The subjects met the following criteria were excluded: abnormal chromosome karyotypes; endometrial abnormalities; severe adenomyosis or hydrosalpinx; PGT cycles; cycles with donor oocyte.

### Subdivisions of study groups

For fresh IVF/ICSI-ET treatment, the participants were divided into three groups. Group 1 (male carrier) included the couples where only male was CPs carrier but female was not; group 2 (female carrier) meant the couples that only female was CPs carrier but male was not; group 3 referred to the couples that neither male nor female were CPs carriers. Accordingly, for FET treatment, all cycles were divided into three groups: group 4 (male carrier), group 5 (female carrier), and group 6 (both male and female without CPs carrier).

### Ovarian stimulation and embryo transfer

The ovarian stimulation protocol was performed as described in our previous studies^16^. GnRH-a 0.05–0.10 mg was administered daily from 7 days after ovulation and lasted for 14–16 days (the long protocol), or injected at day 2 to day 8 of menstrual cycle (for modified short protocol). In the GnRH-a long protocol, leuprorelin acetate 3.75 mg was injected on day 2–5 of the menstrual cycle, and Gn was injected 30–35 days later. In the ultra-long protocol, leuprorelin 3.75 mg was administrated every 28 days and gave 2–3 times, and Gn was injected 21 days after the last injection.

Follicular development and endometrium were monitored with transvaginal ultrasounds. Once there were 3 follicles diameter ≥ 17 mm, women were triggered with 6000–10,000 IU hCG, and oocyte retrieval was performed 36 h later. IVF or ICSI was performed according to sperm quality. No more than two embryos were transferred 72 h after oocyte retrieval.

### Endometrium preparation protocol for FET

The endometrium preparation was performed as described previously^17,18^. In the natural cycle (NC) FET, the dominant follicle and endometrium were monitored from the day 10 of menstrual cycle. No more than 2 frozen-thaw embryos were transferred 3 or 5 days after ovulation. In the hormone replacement treatment (HRT) cycle, estradiol valerate was given from day 3 and last at least 12 days. Progesterone was administered if the endometrial thickness was ≥ 7 mm, and one or two embryos were transferred 3 or 5 days after progesterone administration. In the ovulation induction cycles, letrozole was given from day 3 to day 7, and 37.5 IU-75 IU HMG was administrated if necessary. 10,000 IU hCG was injected for trigger. One or two embryos were transferred 5 days after ovulation.

### Outcomes

The primary outcomes were live birth and clinical pregnancy, and the secondary outcomes were embryo implantation, and early miscarriage. Clinical pregnancy was confirmed by ultrasound visualization of a gestational sac 28–35 days after embryo transfer. Early miscarriage was defined as pregnancy loss before 12 gestational weeks.

### Statistical analysis

All data were analyzed with SPSS 25.0. Continuous data were showed as the mean ± standard deviation (SD), and a Student’s t-test was performed. Categorical data were described as frequencies or percentages and analyzed using a Chi-square test. The association of clinical pregnancy and live birth with cycle characteristic were analyzed by multivariate logistic regression analysis. *P* < 0.05 was set to be statistically significant.

### Ethics approval and consent to participate

The ethical committee of Xiangya Hospital, Central South University approved this study, and the study was conducted according to the Declaration of Helsinki.

## Results

For fresh IVF/ICSI-ET, 10400 fresh IVF/ICSI-ET cycles were enrolled, including 425 male CPs carriers (group 1), 262 female CPs carriers and 9713 non-carriers (group 3). For FET, 11467 FET cycles were included in the present study, in which there were 298 male CPs carriers (group 4), 311 female CPs carriers (group 5), and 10,858 non-carriers (group 6).

The baseline characteristics of eligible subjects were showed in Tables 1 and 2. For fresh IVF/ICSI-ET cycles, the age of women (29.46 ± 4.13 vs. 30.03 ± 3.93 vs. 30.74 ± 4.12, *P* = 0.000), total dosage of Gn (2118.81 ± 767.98 vs. 1900.52 ± 781.09 vs. 2106.36 ± 844.19, *P* = 0.000), duration of COS (11.13 ± 2.64 vs. 10.42 ± 2.56 vs. 10.94 ± 2.73, *P* = 0.003), no. of top-quality embryo (3.66 ± 2.34 vs. 3.40 ± 2.70 vs. 3.92 ± 2.67, *P* = 0.001), no. of embryo transferred (1.78 ± 0.42 vs. 1.85 ± 0.36 vs. 1.88 ± 0.33, *P* = 0.000), P level on trigger day (0.71 ± 0.36 vs. 0.77 ± 0.38 vs. 0.76 ± 0.38, *P* = 0.018) were different among groups. For FET cycles, female age (30.44 ± 4.10 vs. 30.70 ± 4.08 vs. 31.32 ± 4.11, *P* = 0.000), BMI (21.85 ± 2.50 vs. 21.49 ± 2.65 vs. 21.89 ± 2.53, *P* = 0.019), and the no. of embryo transferred (1.74 ± 0.44 vs. 1.76 ± 0.43 vs. 1.80 ± 0.40, *P* = 0.016) were significantly different among groups.

**Table 1:** Characteristic of women undergoing IVF/ICSI-ET cycles.

	Group 1 (n = 425)	Group 2 (n = 262)	Group 3 (n = 9713)	*P* value		
				1 versus 2	1 versus 3	2 versus 3
Age (year)	29.46 ± 4.13	30.03 ± 3.93	30.74 ± 4.12	0.078	0.000	0.006
Duration of infertility	4.36 ± 3.17	4.32 ± 3.18	4.60 ± 3.18	0.890	0.132	0.172
BMI	22.29 ± 2.65	21.80 ± 2.28	22.08 ± 2.54	0.014	0.096	0.078
bFSH	6.40 ± 1.42	6.48 ± 1.54	6.53 ± 1.36	0.444	0.045	0.531
PRL	21.51 ± 16.71	24.08 ± 43.51	22.69 ± 38.62	0.391	0.532	0.561
Total of Gn	2118.81 ± 767.98	1900.52 ± 781.09	2106.36 ± 844.19	0.001	0.765	0.000
Duration of COS	11.13 ± 2.63	10.42 ± 2.56	10.94 ± 2.73	0.001	0.171	0.002
No. of oocyte retrieval	10.92 ± 4.87	10.10 ± 5.22	10.72 ± 4.71	0.026	0.387	0.035
No. of 2PN	7.48 ± 4.09	6.77 ± 4.13	7.18 ± 3.69	0.016	0.106	0.08
No. of top-quality embryo	3.66 ± 2.34	3.40 ± 2.70	3.92 ± 2.67	0.212	0.047	0.002
No. of embryo transferred	1.78 ± 0.42	1.85 ± 0.36	1.88 ± 0.33	0.009	0.000	0.165
EMT	10.87 ± 2.23	10.73 ± 2.26	10.88 ± 2.21	0.424	0.898	0.270
E2 on trigger day	3023.02 ± 1767.02	2968.36 ± 1745.57	2988.76 ± 1768.26	0.694	0.696	0.854
LH on trigger day	1.81 ± 1.46	1.90 ± 1.58	1.58 ± 1.26	0.376	0.049	0.008
P on trigger day	0.71 ± 0.36	0.77 ± 0.38	0.76 ± 0.38	0.024	0.006	0.503

**Table 2:** Characteristic of women undergoing FET cycles.

	Group 4 (n = 298)	Group 5 (n = 311)	Group 6 (n = 10,858)	*P* value		
				4 versus 5	4 versus 6	5 versus 6
Age	30.44 ± 4.10	30.70 ± 4.08	31.32 ± 4.11	0.433	0.000	0.009
Duration of COS	4.95 ± 3.56	4.59 ± 11.02	4.98 ± 3.78	0.277	0.921	0.103
BMI	21.85 ± 2.50	21.49 ± 2.65	21.89 ± 2.53	0.074	0.784	0.005
No. of embryo transferred	1.74 ± 0.44	1.76 ± 0.43	1.80 ± 0.40	0.531	0.015	0.111

As shown in Table 3, the clinical pregnancy rate (55.0% vs. 52.3% vs. 49.7%, *P* = 0.071) and live birth rate (36.9% vs. 37.4% vs. 36.4%, *P* = 0.917) were not significantly different among groups after fresh IVF/ICSI-ET treatment. There were significantly differences in early miscarriage rate (10.7% vs.6.6% vs. 13.3%, *P* = 0.039) and embryo implantation rate (38.8% vs. 36.2% vs. 34.5%, *P* = 0.047) among groups. The clinical pregnancy outcomes after FET were showed in Table 4. There were no significant differences in clinical pregnancy rate (31.8% vs. 30.9% vs. 30%, *P* = 0.667), live birth rate (23.8% vs. 26.3% vs. 23.8%, *P* = 0.486), implantation rate (24.3% vs. 23.6% vs. 22.3%, *P* = 0.326), and early miscarriage rate (12.6% vs. 13.1% vs. 14.4%, *P* = 0.741) among groups.

**Table 3:** Pregnant outcomes between three groups after IVF/ICSI-ET.

	Group 1 (n = 425)	Group 2 (n = 262)	Group 3 (n = 9713)	*P* value
Clinical pregnant rate	55.1% (234/425)	52.3% (137/262)	49.7% (4825/9713)	0.071
Implantation rate	38.8% (293/756)	36.2% (175/484)	34.5% (6296/18,225)	0.047
Miscarriage rate	10.7% (25/234)	6.6% (9/137)	13.3% (642/4825)	0.039
Live birth rate	36.9% (157/425)	37.4% (98/262)	36.4% (3532/9713)	0.917

**Table 4:** Pregnant outcomes between three groups after FET.

	Group 4 (n = 298)	Group 5 (n = 311)	Group 6 (n = 10,858)	*P* value
Clinical pregnant rate	31.8% (139/298)	30.9% (139/311)	30.0% (4653/10,858)	0.667
Implantation rate	24.3% (167/519)	23.6% (169/548)	22.3% (5601/19,533)	0.326
Miscarriage rate	12.6% (20/139)	13.1% (21/139)	14.4% (782/4653)	0.741
Live birth rate	23.8% (93/298)	26.3% (111/311)	23.8% (3387/10,858)	0.486

The association of clinical pregnancy and live birth with cycle characteristics were assessed with logistic regression analysis. For clinical pregnancy after fresh IVF/ICSI-ET (Table 5), CPs carrier (*P* < 0.05), no. of embryo transferred (*P* < 0.01), endometrial thickness (*P* < 0.01), LH (*P* < 0.01), and P level on trigger day (*P* < 0.05) were correlated with clinical pregnancy. For live birth after fresh IVF/ICSI-ET, no. of embryo transferred (*P* < 0.01) and endometrial thickness (*P* < 0.01) were associated with live birth. For FET cycles (Table 6), female age (*P* < 0.01), duration of infertility (*P* < 0.01), and no. of embryo transferred (*P* < 0.01) were associated with clinical pregnancy and live birth.

**Table 5:** Logistic model for clinical pregnancy and live birth after fresh IVF/ICSI-ET.

	Clinical pregnancy			Live birth		
	B	OR[Exp(B)]	*Sig.*	B	OR[Exp(B)]	*Sig.*
Group	/	/	0.015	/	/	0.474
Group(1)	0.304	1.355 [1.087, 1.690]	0.007	0.046	1.047 [0.834, 1.314]	0.692
Group(2)	0.159	1.172 [0.889, 1.545]	0.261	0.168	1.183 [0.893, 1.567]	0.242
Age	0.004	1.004 [0.991, 1.016]	0.581	− 0.004	0.996 [0.983, 1.009]	0.533
Duration of infertility	− 0.001	0.999 [0.984, 1.013]	0.857	0.002	1.002 [0.987, 1.017]	0.802
BMI	− 0.006	0.994 [0.977, 1.012]	0.513	− 0.008	0.992 [0.974, 1.010]	0.382
bFSH	0.022	1.022 [0.981, 1.065]	0.290	0.030	1.031 [0.988, 1.075]	0.158
PRL	0.000	1.000 [0.999, 1.001]	0.994	0.000	1.000 [0.999, 1.001]	0.664
Total Gn	0.000	1.000 [1.000, 1.000]	0.329	0.000	1.000 [1.000, 1.000]	0.277
Duration of COS	− 0.006	0.994 [0.974, 1.015]	0.588	− 0.001	0.999 [0.978, 1.021]	0.950
No. of oocyte	0.012	1.012 [0.996, 1.028]	0.152	− 0.006	0.994 [0.978, 1.010]	0.465
No. of 2PN	0.027	1.028 [0.957, 1.104]	0.450	0.051	1.052 [0.978, 1.133]	0.174
No. of cleavage 2PN	− 0.032	0.968 [0.902, 1.040]	0.379	− 0.04	0.961 [0.892, 1.034]	0.285
No. of top-quality embryo	− 0.008	0.992 [0.971, 1.013]	0.440	− 0.006	0.994 [0.972, 1.015]	0.563
No. of embryo transferred	0.845	2.328 [1.970, 2.751]	0.000	0.835	2.306 [1.924, 2.763]	0.000
EMT	0.088	1.092 [1.070, 1.114]	0.000	0.097	1.102 [1.079, 1.124]	0.000
E2 on trigger day	0.000	1.000 [1.000, 1.000]	0.848	0.000	1.000 [1.000, 1.000]	0.897
LH on trigger day	0.064	1.066 [1.030, 1.102]	0.000	0.000	1.000 [0.965, 1.035]	0.983
P on trigger day	− 0.154	0.857 [0.758, 0.970]	0.014	0.008	1.008 [0.887, 1.145]	0.901

**Table 6:** Logistic model for clinical pregnancy and live birth after FET.

	Clinical pregnant			Live birth		
	B	OR[Exp(B)]	*Sig.*	B	OR[Exp(B)]	*Sig.*
Group	/	/	0.428	/	/	0.418
Group(1)	0.145	1.157 [0.917, 1.458]	0.218	− 0.028	0.972 [0.757, 1.249]	0.827
Group(2)	0.054	1.055 [0.839, 1.327]	0.645	0.158	1.171 [0.922, 1.488]	0.195
Age	− 0.019	0.981 [0.971, 0.990]	0.000	− 0.048	0.953 [0.943, 0.963]	0.000
Duration of infertility	− 0.019	0.981 [0.970, 0.993]	0.001	− 0.015	0.985 [0.974, 0.987]	0.012
BMI	0.009	1.009 [0.994, 1.024]	0.234	− 0.009	0.991 [0.975, 1.007]	0.268
No. of embryo transferred	− 0.1551	1.228 [1.118, 1.348]	0.000	0.29	1.336 [1.205, 1.481]	0.000

## Discussion

To the best of our knowledge, this is the first large sample size study (n = 21867) involving fresh cycles and FET cycles to comprehensively explore the effect of CPs on reproductive outcomes of ART. In this retrospective study, we found that CPs have no negative effects on pregnancy outcomes after fresh IVF/ICSI-ET and FET treatment.

CPs was considered to be benign and harmless. However, recently, some researchers have found that the heterochromatin in CP regions might inhibit or silence gene expression through the reversible transformation between heterochromatin and euchromatin^19,20^. Besides, other studies have shown that the heterochromatin located at the centromeres, played a vital role in cell division. Chromatin mutation in these regions may lead to abnormal division of meiotic cell, such as defective in centromeres function and centromere assembly, difficulty in homologous chromosome pairing, and disrupted cell division, which can affect the formation of functional sperm^21^.

The impact of CPs on outcomes after ART was explored by previous studies, but the results were inconclusive and remained controversial. Early in 2005, Yakin et al. enrolled infertile man receiving ICSI treatment, and found that CPR and IR were significantly lower for men with CPs than that without CPs^15^. Similarly, a few of studies^12,14^ suggested that CPs had detrimental effects on spermatogenesis, negatively affected the outcomes of IVF/ICSI-ET treatment. Li et al. reported that individual genetic counseling should be afforded according to the polymorphism types^6^. However, researches by Hong et al. indicated that CPs appeared to have no adverse effects on the outcome of IVF-ET treatment^4,11^, inv(9) in one partner has satisfactory outcomes^10^. There were studies found that couples with CPs in male carriers have poor pregnancy outcomes after IVF-ET, with higher EMR and lower LBR after a complete cycle^13^.

In present study, the female age was younger in groups 1 and 2 than that in group 3. In contrast with previous studies, we did not find that male carrying CPs was associated with outcomes after ART. Our results suggested female carrying CPs led to less 2PN cleavage embryos and top-quality embryos, which might result from less Gn dosage and short duration of COS. Besides, the CPR and LBR were not significantly different among groups, although the no. of embryo transferred in the group 3 was more than that in the rest groups. The EMR was higher in group 3 than that in other groups, and this may partly resulted from the female age.

In FET cycles, the female age was older and the no. of embryos transferred was higher in group 6 than that of groups 4 and 5. The IVF outcomes, including CPR, LBR, IR, and the EMR, were all similar among groups after FET treatment. The results of our study were inconsistent with previous studies indicating that the risk of miscarriage increased when the male partner had a large Y chromosome^22,23^. Then, using a logistic regression model, we found that no. of embryo transferred and endometrial thickness were associated with outcomes after fresh IVF/ICSI-ET. And female age, duration of infertility, and no. of embryo transferred were related with outcomes after FET treatment. CPs seems have no effects on outcomes after embryo transfer.

The strength of this study was its large sample size involving 21,867 transfer cycles. On the whole, CPs had no significant negative effects on the clinical outcomes of ART treatment. In addition, the present study firstly assessed the impact of CPs on FET outcomes. The results of the present study indicated the CPs have no negative impacts on the embryo quality and endometrial receptivity. At last, the live birth rate was followed-up, and defined as the primary outcome.

Certainly, this study has a few limitations. Firstly, it was a retrospective study, and the potential heterogeneity and possible confounding factors were inevitable. Besides, we did not stratified infertility according to the specific type of CPs, and subject carrying any type of CPs was considered as a carrier. Despite these drawbacks, this study reflected a real state without intervention in the real world.

## Conclusion

The clinical pregnancy and live birth of IVF/ICSI-ET and FET treatment did not appear to be adversely affected by CPs. Only female CPs carrier led to lower 2PN cleavage rate. In addition, the chromosome analysis method in this study had a band resolution of 400–550 BPHS; therefore, it was difficult to distinguish some potential variations from common polymorphism variations. Therefore, further research with more sensitive techniques is needed.

## Data Availability

The datasets supporting the conclusions of this article are included within the article.

## Abbreviations

ART: Assisted reproductive technology
IVF: In vitro fertilization
ICSI: Intracytoplasmic sperm injection
FET: Frozen-thawing embryo transfer
PGT: Preimplantation genetic testing
CPs: Chromosomal polymorphisms
